# Early post-treatment ^18^F-FDG PET/CT for predicting radiation-induced hypothyroidism in head and neck cancer

**DOI:** 10.1186/s40644-022-00494-y

**Published:** 2022-10-10

**Authors:** Hsi-Huei Lu, Nan-Tsing Chiu, Mu-Hung Tsai

**Affiliations:** 1 Division of Nuclear Medicine, Department of Medical Imaging, College of Medicine, National Cheng Kung University Hospital, National Cheng Kung University, Tainan, Taiwan; 2 Department of Radiation Oncology, College of Medicine, National Cheng Kung University Hospital, National Cheng Kung University, No. 138 Sheng Li Rd, Tainan, Taiwan

**Keywords:** Radiation-induced hypothyroidism, ^18^F-FDG PET/CT, Head and neck cancer

## Abstract

**Background:**

Radiation-induced hypothyroidism (RIHT) is a common, but underestimated, late adverse effect in head and neck cancer. We investigated the value of early post-treatment ^18^F-fluorodeoxyglucose (FDG) positron emission tomography/computed tomography (PET/CT) for predicting RIHT.

**Methods:**

We searched our institutional database for patients aged ≥ 20 years who had undergone definitive radiotherapy for nasopharyngeal or oropharyngeal cancer between 2005 and 2017, followed by ^18^F-FDG PET/CT within 180 days of radiotherapy completion. We visually assessed and compared PET/CT and baseline characteristics in patients with and without RIHT using the chi-square test for categorical variables and the *t*-test for continuous variables. Variable predictive ability was evaluated by measuring the area under receiver operating characteristic curves.

**Results:**

Fifty-two patients were included; 22 (42%) developed RIHT and 30 (58%) did not. Two patients presented with diffuse thyroid uptake on PET/CT via visual assessment, and both developed RIHT later. Among the PET/CT variables, thyroid functioning volume was significantly higher in patients without RIHT than in patients with RIHT (16.30 ± 6.03 cm^3^ vs. 10.61 ± 3.81 cm^3^, p < 0.001). The maximum standard uptake values of the thyroid and pituitary glands did not differ significantly between the groups. Two patient characteristics, pretreatment thyroid volume and mean radiotherapy dose to the thyroid, also showed significant differences between the groups. An algorithmic approach combining visual grading of thyroid ^18^F-FDG uptake and thyroid functioning volume cutoff of 14.01 yielded an area under curve of 0.89 (95% confidence interval, 0.80–0.98); the sensitivity, specificity, positive predictive value, and negative predictive value were 87.0%, 82.3%, 80.0%, and 88.9%, respectively.

**Conclusion:**

Early post-treatment PET/CT-derived thyroid functioning volume was a good predictor of RIHT development. Diffusely increased thyroid ^18^F-FDG uptake on PET/CT may indicate impending RIHT. Routine surveillance of thyroid function is warranted in patients at high risk of developing RIHT.

## Introduction

Head and neck cancers are diverse histopathologic malignancies that arise in the oral cavity, pharynx, larynx, salivary glands, and sinuses, with geographic disparities. Radiation therapy plays a key role in their curative-intent treatment, particularly in cancers with nasopharyngeal and oropharyngeal origins. Intensity-modulated radiation therapy allows tumor dose escalation through computer-assisted systems that generate dose distributions that conform to the target volume while minimizing the dose to nearby normal tissues. Radiotherapy-induced hypothyroidism (RIHT) is the most common thyroid disorder in recipients of neck irradiation, with a reported incidence of 20–50% [1–3]. RIHT usually develops several months to years after the completion of radiotherapy.

The ^18^F-fluorodeoxyglucose (FDG) positron emission tomography (PET)/computed tomography (CT) plays an important role in the management of malignancies. For head and neck cancer, it provides additional value to conventional imaging in staging, risk stratification, and response assessment after definitive radiotherapy; it also provides valuable information on distant metastasis [4–6]. A prospective, randomized, controlled trial showed that post-treatment PET/CT-guided surveillance is non-inferior to and more cost-effective than routine neck dissection for head and neck cancers with advanced nodal status [7]. Post-treatment PET/CT is often employed to aid decision making in challenging scenarios, such as those in which plasma Epstein–Barr virus DNA levels are persistently elevated after definitive therapy or in which diagnosis of a residual tumor from inflammation via conventional magnetic resonance imaging cannot be made with confidence.

Radiation is a recognized iatrogenic cause of hypothyroidism, but RIHT remains underdiagnosed owing to overlooked symptoms and lack of a follow-up consensus. Physiologically normal thyroids generally show little or no ^18^F-FDG uptake on PET/CT scans. Incidental diffuse ^18^F-FDG uptake in the thyroid has been reported in 0.6–3.3% of the population and is mainly related to thyroiditis or hypothyroidism [8]. Interim or end-of-treatment PET/CT has been used to predict hypothyroidism in patients treated with immunotherapy [9–12]; however, the prediction of RIHT using PET/CT has not been previously reported. In this study, we aimed to investigate the value of early post-treatment ^18^F-FDG PET/CT for predicting RIHT.

## Materials and methods

### Study design and patient selection

This study included patients treated at a tertiary academic medical center between 2005 and 2017. We retrospectively searched our institutional database for patients aged ≥ 20 years who had undergone ^18^F-FDG PET/CT within 180 days from the date of completion of definitive radiotherapy for nasopharyngeal or oropharyngeal cancer. Exclusion criteria were a history of surgery or radiation therapy to the neck, radical surgery or neck dissection as part of the primary treatment, presence of baseline thyroid function abnormalities, presence of thyroid nodules on available exams, or no thyroid function follow-up. The study was approved by the Institutional Review Board of National Cheng Kung University Hospital, and the requirement of informed consent was waived due to the retrospective nature of the study.

### Treatment and follow-up

Patients were treated in accordance with the institutional guidelines. Definitive radiotherapy was prescribed to at least 70Gy using linear accelerators. Neoadjuvant or concurrent chemotherapy was administered at the discretion of the treating physician. There were no institution-wide dose constraints for the thyroid.

In accordance with local guidelines, patients were followed up every 1–3 months in the first year, every 2–6 months in the second year, and every 4–8 months in the third to fifth year after treatment, and every 12 months thereafter. Serial monitoring of thyroid function was recommended every 6–12 months for definitive radiotherapy recipients. Thyroid function was evaluated using chemiluminescence immunoassay (CLIA) or radioimmunoassay (RIA). Hypothyroidism was defined as an elevated thyroid-stimulating hormone level (TSH; reference institutional range for CLIA, 0.27–4.20 U/mL; range for RIA, 0.25–4.00 U/mL) or abnormally low free thyroxine (T4) level (reference institutional range for CLIA, 0.93–1.70 ng/dL; range for RIA, 0.89–1.79 ng/dL). Patients with high TSH or low T4 levels were referred to an endocrinologist for potential thyroxine supplementation.

### ^18^F-FDG PET/CT imaging

Before each examination, patients only received water for at least 6h with confirmed serum glucose levels lower than 200mg/dL. The ^18^F-FDG (370 MBq) was injected intravenously with the patient in the supine position. Images from the head to upper thighs were acquired 60min after injection using a PET/CT scanner (Biograph 6 or mCT Flow; Siemens, Munich, Germany). Before each PET scan, a preceding low-dose non-contrast-enhanced CT was performed with a pitch of 0.8, voltage of 100 to 130kV, auto mA based on topogram, matrix size 512 and slice thickness of 3.0mm for attenuation correction. PET scan was acquired with an acquisition time of 3min per bed and reconstructed with a standard order-subset expectation maximization algorithm with 2 iterations and 21 subsets, image size 200, Gaussian filter, and full width at half maximum of 5.0mm.

### ^18^F-FDG PET/CT analysis

**Fig. 1:**
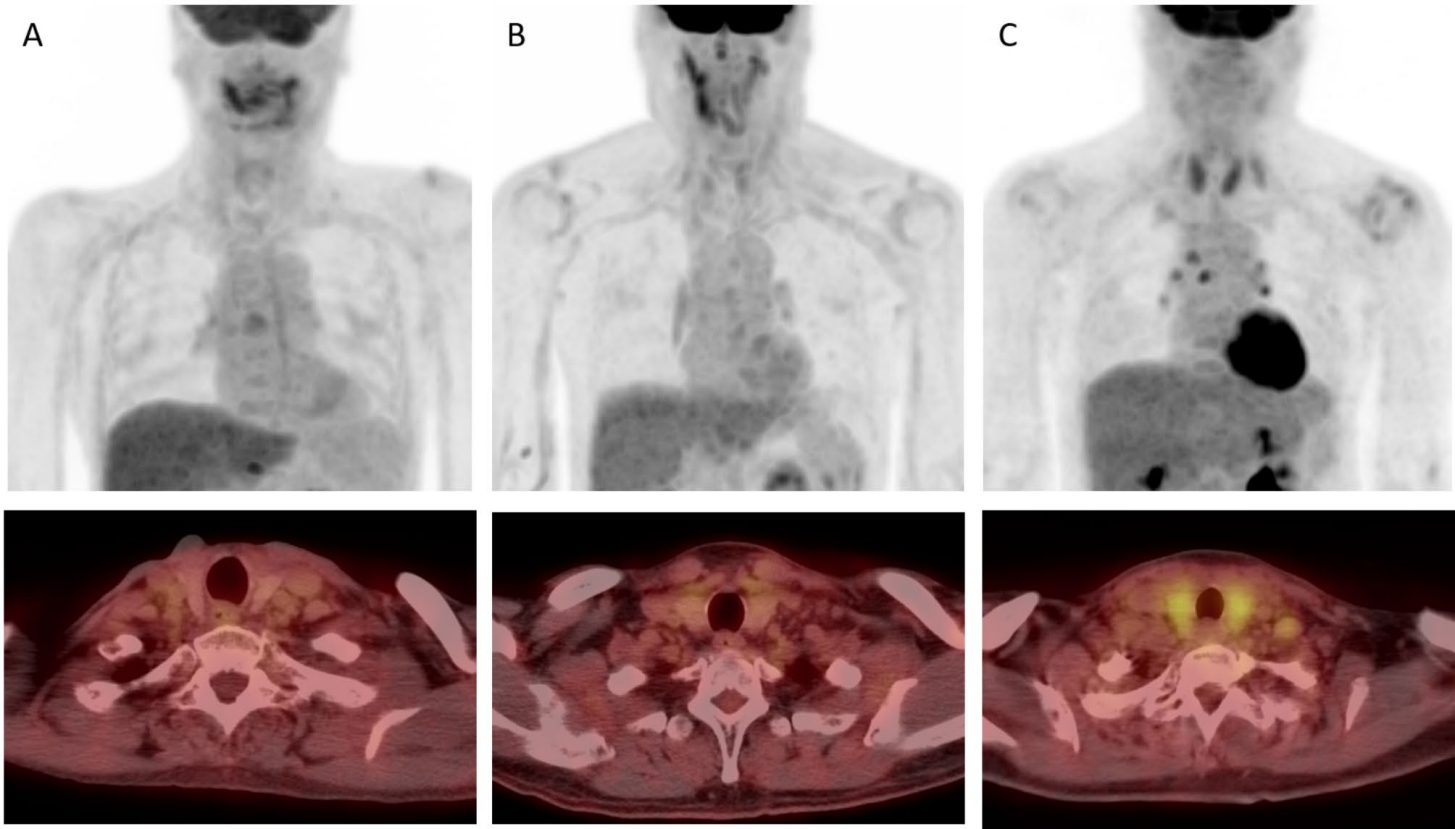
Examples of thyroid visual grade in our cohort (Upper: maximum intensity projection, lower: transaxial view). **A** Grade 1, thyroid uptake less than blood pool uptake; **B** grade 2, thyroid uptake equal to or higher than blood pool uptake but less than liver uptake; **C** grade 3, thyroid uptake equal to or higher than liver uptake

The degree of radioactivity in the thyroid was first determined by visual grading: grade 1, thyroid uptake less than mediastinal blood pool uptake; grade 2, thyroid uptake similar to or higher than blood pool uptake but less than liver uptake; grade 3, thyroid uptake similar to liver uptake; and grade 4, thyroid uptake greater than liver uptake (Fig.1). Diffuse thyroid uptake was defined as visual grade ≥ 3.

Semi-quantitative parameters were analyzed using PET/CT software (syngo.via; Siemens). Three-dimensional regions of interest (ROIs) were drawn separately around the left and right thyroid lobes to obtain the maximum and mean standard uptake values (SUVmax and SUVmean, respectively). Thyroid functioning volume was defined as the summed volume of bilateral thyroid glands segmented on PET scan using a 40% threshold of SUVmax; the registrated CT image is referenced when thyroid uptake on PET scan is difficult to identify. The SUVmax of the pituitary gland and blood pool was obtained by measuring ^18^F-FDG uptake in the pituitary fossa and aortic arch, respectively.

### Thyroid volume and dose parameters

We queried the radiotherapy treatment planning system to obtain the pretreatment parameters for the thyroid. The volume of the contoured thyroid was calculated. The mean thyroid radiation dose was retrieved from the radiotherapy plan.

### Statistical analysis

The development of hypothyroidism was the primary binary outcome. We compared the baseline characteristics and PET/CT parameters of patients with and without RIHT using the chi-square test for categorical variables and *t*-test for continuous variables. Variable predictive ability was evaluated by generating a receiver operating characteristic (ROC) curve and calculating the area under the curve (AUC). ROC curves were compared using the DeLong method. Internal validation was performed via bootstrap sampling of 10,000 samples with repeated sampling. A p < 0.05 was considered to be statistically significant. Statistical analysis was performed using R software version 4.0.4 (R Foundation for Statistical Computing, Vienna, Austria).

## Results

### Patient characteristics

We screened 460 patients who met the inclusion criteria. Of these patients, 185 underwent PET/CT after completion of definitive radiotherapy. Restricting the timing of the PET/CT scan to within 180 days of treatment completion yielded 55 patients. Three patients were excluded owing to the presence of a thyroid nodule, difficulty in determining the thyroid ROI due to proximity with nearby malignant lesions, and exam conducted elsewhere; thus, the final number of patients eligible for analysis was 52. Most of the patients were men (75%) and had nasopharyngeal cancer (85%), T3–T4 disease (62%), and N2–N3 disease (81%). The median prescribed radiation dose was 72Gy (Table1).

**Table 1:** Patient baseline characteristics (n = 52)

Characteristic	Value
Age (years), median (IQR)	52.6 (46.3–60.2)
Sex, n (%)	
Male	39 (75)
Female	13 (25)
Diagnosis, n (%)	
Nasopharyngeal cancer	44 (84.6)
Oropharyngeal cancer	8 (15.4)
Clinical T classification, n (%)	
T1	6 (11.5)
T2	14 (26.9)
T3	12 (23.1)
T4	20 (38.5)
Clinical N classification, n (%)	
N0	1 (1.9)
N1	9 (17.3)
N2	30 (57.7)
N3	12 (23.1)
Prescribed radiotherapy dose (Gy), median (IQR)	72 (70–74)
Pretreatment thyroid volume (cm^3^), mean (SD)	15.4 (6.5)
Thyroid dose (Gy), median (IQR)	44.1 (37.4–50.4)
Pituitary dose (Gy), median (IQR)	50.3 (23.6–65.1)
Time from end of radiotherapy to PET (days), median (IQR)	93 (77–112)
RIHT, n (%)	
Yes	20 (42)
No	30 (58)
Time from end of radiotherapy to RIHT (months), median (IQR)	22.7 (16.4–25.2)

Abbreviations: IQR, interquartile range; SD, standard deviation; PET, positron emission tomography; RIHT, radiotherapy-induced hypothyroidism

**Fig. 2:**
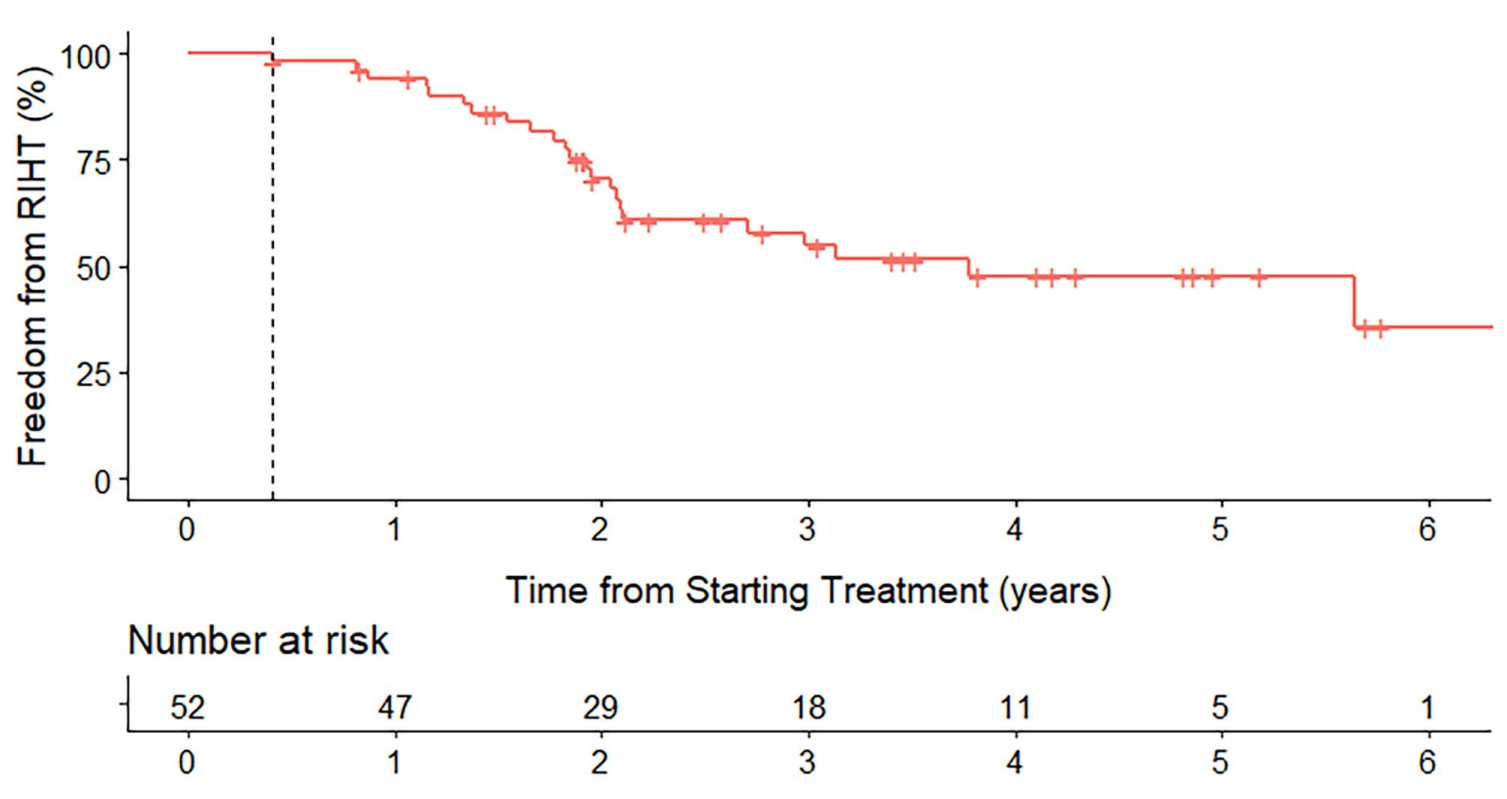
Freedom from radiation-induced hypothyroidism (RIHT) in our cohort. The vertical dashed line indicates the median time when post-treatment positron emission tomography was performed

The median time to PET/CT from completion of radiotherapy was 93 (interquartile range [IQR], 76–112) days. The median thyroid function follow-up period was 3.3 (IQR, 1.8–4.7) years. Twenty-two patients (42%) developed RIHT, whereas 30 (58%) did not. No patient had abnormal thyroid function before PET/CT. The median time to RIHT was 22.7 (IQR, 16.4–25.2) months after treatment (Fig.2) and 17.6 (IQR 12.0–20.4) months after PET/CT.

### Variables related to RIHT

First, we visually assessed thyroid uptake on PET/CT. Two patients (4%) had diffuse thyroid uptake (visual grade 3) on PET/CT, whereas 32 (62%) and 18 (35%) had visual grade 2 and 1 uptake, respectively. The two patients with visual grade 3 uptake later developed RIHT.

Of the PET/CT-derived factors, the thyroid functioning volume was significantly higher in patients without RIHT than in patients with RIHT (16.30 ± 6.03 vs. 10.61 ± 3.81 cm^3^, p < 0.001) (Table2). There was no significant difference in other PET/CT-derived factors, including the SUVmax for the thyroid and pituitary glands, between the two groups. In addition, two factors differed significantly between patients without and with RIHT: pretreatment thyroid volume (17.9 ± 6.6 vs. 12.1 ± 4.7 cm^3^, p = 0.001) and mean radiotherapy dose to the thyroid (40.9 ± 10.6 vs. 48.6 ± 8.4, p = 0.007). There was no significant difference in mean radiotherapy dose to the pituitary gland.

**Table 2:** Patient and PET parameters for the normal thyroid (n = 30) and hypothyroid (n = 22) groups

Characteristic	Normal	Hypothyroid	*p* value
*Patient factor*			
Age (years)	53.6 (9.6)	50.0 (10.1)	0.209
Male sex, n (%)	23 (76.7%)	16 (72.7%)	1.000
Pretreatment thyroid volume (cm^3^)	17.9 (6.6)	12.1 (4.7)	0.001 *
Mean radiotherapy dose to thyroid (Gy)	40.9 (10.6)	48.6 (8.4)	0.007 *
Mean radiotherapy dose to pituitary (Gy)	42.8 (24.6)	43.4 (27.6)	0.937
*Early post-treatment PET parameter*			
Thyroid SUVmax	2.57 (1.41)	2.35 (0.69)	0.511
Thyroid SUVmean	1.89 (0.87)	1.80 (0.45	0.680
Thyroid TLG	14.10 (8.27)	11.04 (7.09)	0.167
Thyroid functioning volume, cm^3^	16.30 (6.03)	10.61 (3.81)	< 0.001 *
Blood pool SUVmax	2.10 (0.41)	2.06 (0.36)	0.688
Thyroid SUVmax/blood pool SUV ratio	1.15 (0.20)	1.17 (0.32)	0.752
Pituitary SUVmax	2.76 (0.54)	2.81 (0.46)	0.712
Pituitary SUVmean	2.38 (0.48)	2.36 (0.34)	0.861

Abbreviations: PET, positron emission tomography; SUV, standardized uptake value; TLG, total lesion glycolysis. *, p < 0.05

**Fig. 3:**
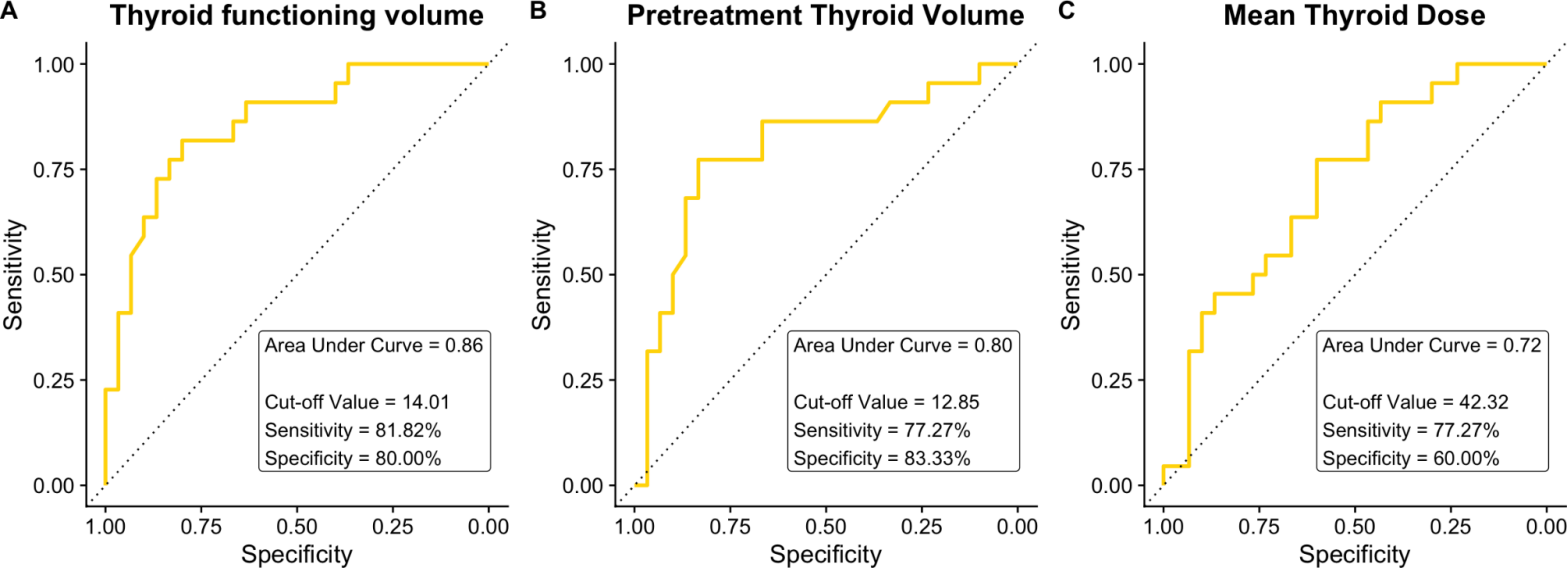
Receiver operating characteristic curves for **A** early post-treatment positron emission tomography-derived thyroid functioning volume, **B** pretreatment thyroid volume, and **C** mean thyroid dose

ROC curve analysis of our cohort showed that post-treatment thyroid functioning volume (AUC, 0.86; 95% confidence interval [CI], 0.76–0.96), pretreatment thyroid volume (AUC, 0.80; 95% CI, 0.67–0.93), and mean radiotherapy dose to the thyroid (AUC, 0.72; 95% CI, 0.58–0.86) had good discriminating ability for prediction of late-onset RIHT (Fig.3). Thyroid functioning volume tended to have greater AUC than pretreatment thyroid volume and mean thyroid dose (p > 0.05). Internal validation via bootstrapping with 10,000 samples yielded similar performances: the 95% CIs of the AUCs for thyroid functioning volume, pretreatment thyroid volume, and mean radiotherapy dose to the thyroid were 0.75–0.95, 0.66–0.92, and 0.57–0.85, respectively. Using a thyroid functioning volume cutoff value of 14.01, the sensitivity, specificity, positive predictive value, and negative predictive value for the development of RIHT were 81.8%, 80.0%, 75.0%, and 85.7%, respectively. An algorithmic approach combining visual grading and thyroid functioning volume cutoff value yielded an AUC of 0.89, with a 95% CI of 0.80–0.98; the sensitivity, specificity, positive predictive value, and negative predictive value were 87.0%, 82.3%, 80.0%, and 88.9%, respectively.

## Discussion

In this study, visual grading of thyroid uptake combined with thyroid functioning volume derived from early post-treatment PET/CT significantly correlated with the development of RIHT in patients with head and neck cancer treated with definitive radiotherapy. Moreover, the date of early post-treatment PET/CT preceded the appearance of abnormal thyroid function by a median of 1.5 years.

The incidence of RIHT gradually increases with time; it is less than 10% at 6 months and up to 50% at 3 years post-treatment [13]. The incidence of RIHT in our study (42%) is consistent with the literature findings. RIHT is often insidious but is easily neglected because it is usually subclinical or asymptomatic; however, prolonged insufficiency of thyroid hormones can ultimately lead to cardiac and cognitive dysfunction [14]. Although screening for RIHT is recommended after head and neck radiotherapy, the optimal screening interval remains unknown. In patients with low risk, infrequent screening at intervals of 1–2 years may be adequate, while scrupulous screening every 3–6 months may be required for high-risk patients. However, despite several risk factors of RIHT have been identified, accurate prediction of RIHT risk has not been achieved.

PET/CT is widely used in the field of oncology, particularly for evaluating response after treatment. In locally advanced head and neck cancers, it is the preferred modality for response evaluation after radiotherapy. Therefore, most patients are likely to receive PET/CT as part of their standard care. Although the study is performed primarily to evaluate tumor response, it also provides an added opportunity to improve risk prediction of RIHT. Our study focused on early (within 180-days post-irradiation) PET/CT as a predictor of RIHT and was unique in that thyroid function (as indicated by laboratory values) was normal at the time of PET/CT.

Several clinical parameters, including age, sex, pre-existing thyroid disease, thyroid size, and radiation dose, have been associated with the development of RIHT. A prospective study identified mean radiotherapy dose to the thyroid and pretreatment thyroid volume as potential predictors of RIHT development [15]; these predictors were validated in subsequent studies on breast cancer and nasopharyngeal carcinoma [16, 17], as well as in the present study. Interestingly, in the present study, the AUCs for these predictors were lower (although not significantly) than those for early post-treatment thyroid functioning volume. Trends in thyroid volume change after radiation have been reported by Lin et al. [18, 19], who found that thyroid volume dramatically decreased (up to 20%) in the first 6 months after radiotherapy and partially recovered after 30 months. Moreover, thyroid volume reduction correlated with the mean radiation dose received by the thyroid, suggesting that direct radiation-induced damage to the thyroid cells and vessels may have led to volume shrinkage. Unlike pretreatment thyroid volume and mean dose, thyroid functioning volume may represent sublethal injury to the thyroid, possibly resulting in the higher predictive ability of RIHT in our study.

On ^18^F-FDG PET/CT scans, normal thyroids usually show low amounts of homogenously distributed radioactivity, with uptake less than or approximately equal to that of the blood pool (visual grade 1–2) [20]. Focal ^18^F-FDG -avid thyroid lesions are potentially malignant, whereas lesions with diffusely increased thyroid ^18^F-FDG uptake are associated with thyroid disorders, such as thyroiditis or hypothyroidism [21]. ^18^F-FDG uptake has been shown to correlate with the development of hypothyroidism in patients treated with immunotherapy agents [11, 12]; thyroid dysfunction due to immune-mediated damage is the most common immune-related endocrinological adverse event, with a reported incidence of 7–21% [22, 23]. In patients treated with immunotherapy, the SUVmax and SUVmean of the thyroid in the end of treatment PET/CT were significantly higher in patients who developed immunotherapy-related thyroiditis than in those who did not [11, 12]. However, these parameters failed to correlate with the development of RIHT in the present study. This discrepancy can be attributed to differences in the interval between the PET/CT scan and onset of RIHT vs. immunotherapy-induced thyroiditis. The development of immunotherapy-induced thyroiditis is relatively rapid, with a mean time of 6 (IQR 3–8) weeks between the start of the treatment and its detection [23]. In contrast, development of RIHT is usually delayed, with peak occurrence at 2–3 years after radiation [24].

Hypothyroidism-related pathological thyroid uptake on PET/CT may occasionally be the first sign of hypothyroidism, even preceding serum irregularities. In a study of hypothyroidism caused by immune checkpoint inhibitors, diffusely increased thyroid ^18^F-FDG uptake preceded or coincided with abnormal serum function in 5 of 7 (71.4%) patients who eventually developed hypothyroidism [23]. The two patients who had diffuse thyroid ^18^F-FDG uptake in our study both subsequently developed RIHT. Incorporation of the visual grade of thyroid has the potential to further improve the predictive ability than using thyroid functioning volume alone. Careful symptom monitoring and close surveillance of thyroid function are warranted in patients with diffuse thyroid ^18^F-FDG uptake, even in those with seemingly normal thyroid function.

In addition to direct injury to the thyroid glands, RIHT may also cause damage to the pituitary gland via the hypothalamus–pituitary–thyroid axis, known as central hypothyroidism. It is a concern mostly in patients with nasopharyngeal carcinoma who receive a considerable dose of radiation to the pituitary gland owing to its proximity to the nasopharynx radiation field. The main predictive factor for the development of central hypothyroidism is the pituitary radiation dose. In the present study, neither the mean radiation dose to the pituitary gland nor the pituitary PET/CT parameters correlated with RIHT development, possibly because of the low incidence [25] and long median latency period (4.8 years) [26] of central hypothyroidism.

The main limitation of the present study was its retrospective design and relatively small sample size; a larger sample size may have had sufficient power to show additional significant differences. As RIHT may occur years after treatment, it is possible that some patients developed RIHT after the follow-up period; these patients would be erroneously classified as not having RIHT. Owing to a lack of data, no external validation was performed in the study, and generalization of results should be exercised with caution. Despite these limitations, we showed that incorporation of thyroid parameters from early post-treatment PET/CT improves ability to predict RIHT.

## Conclusion

In this study, we found early post-treatment PET/CT-derived thyroid functioning volume was a good predictor of RIHT development; diffusely increased thyroid ^18^F-FDG uptake on PET/CT may indicate impending RIHT. In patients with these high-risk findings, vigilant surveillance of thyroid function is warranted.

## Data Availability

The research dataset for the current study are available from the corresponding author upon reasonable request.

## Abbreviations

RIHT: Radiation-induced hypothyroidism
FDG: Fluorodeoxyglucose
PET/CT: Positron emission tomography/Computed tomography
TSH: Thyroid-stimulating hormone
T4: Thyroxine
ROI: Region of interest
ROC: Receiver operating characteristic
AUC: Area under the curve
IQR: Interquartile range
